# Reference exome data for Australian Aboriginal populations to support health-based research

**DOI:** 10.1038/s41597-020-0463-1

**Published:** 2020-04-29

**Authors:** Alexia L. Weeks, Heather A. D’Antoine, Melita McKinnon, Genevieve Syn, Dawn Bessarab, Ngiare Brown, Steven Y. C. Tong, Bo Reményi, Andrew Steer, Lesley-Ann Gray, Michael Inouye, Jonathan R. Carapetis, Jenefer M. Blackwell, Timo Lassmann

**Affiliations:** 1 Telethon Kids Institute, The University of Western Australia, Perth Children’s Hospital, Perth, Western Australia Australia; 2 Menzies School of Health Research, Charles Darwin University, Darwin, Northern Territory Australia; 3 Centre for Aboriginal Medical and Dental Health, The University of Western Australia, Crawley, Western Australia; 4 School of Education, The University of Wollongong, New South Wales, Australia; 5 Victorian Infectious Disease Service, The Royal Melbourne Hospital, and Doherty Department, The University of Melbourne, at the Peter Doherty Institute for Infection and Immunity, Victoria, Australia; 6 Group A Streptococcal Research Group, Murdoch Childrens Research Institute, Melbourne, Victoria, Australia and Centre for International Child Health, Department of Paediatrics, Royal Children’s Hospital, Melbourne, Victoria Australia; 7 Baker Heart and Diabetes Institute, Melbourne, Victoria Australia; 8 Department of Public Health and Primary Care, The University of Cambridge, Cambridge, UK

**Keywords:** Genetics, Genetics research

## Abstract

Whole exome sequencing (WES) is a popular and successful technology which is widely used in both research and clinical settings. However, there is a paucity of reference data for Aboriginal Australians to underpin the translation of health-based genomic research. Here we provide a catalogue of variants called after sequencing the exomes of 50 Aboriginal individuals from the Northern Territory (NT) of Australia and compare these to 72 previously published exomes from a Western Australian (WA) population of Martu origin. Sequence data for both NT and WA samples were processed using an ‘intersect-then-combine’ (ITC) approach, using GATK and SAMtools to call variants. A total of 289,829 variants were identified in at least one individual in the NT cohort and 248,374 variants in at least one individual in the WA cohort. Of these, 166,719 variants were present in both cohorts, whilst 123,110 variants were private to the NT cohort and 81,655 were private to the WA cohort. Our data set provides a useful reference point for genomic studies on Aboriginal Australians.

## Abstract (summary)

**Table:** 

Measurement(s)	Aboriginal Australian • DNA • sequence feature annotation
Technology Type(s)	Whole Exome Sequencing • DNA sequencing • sequence annotation
Factor Type(s)	ancestry • sex • age
Sample Characteristic - Organism	Homo sapiens
Sample Characteristic - Location	Northern Territory

Machine-accessible metadata file describing the reported data: 10.6084/m9.figshare.12040638

## Background & Summary

Whole exome sequencing (WES) is a popular and successful technology which is becoming more widely used in both research and clinical settings^1,2^. This technology is a fraction of the cost of other methods such as whole genome sequencing (WGS) and provides informative data on common variants found widely in the general population, as well as novel variants and variants involved in genetic diseases^1^.

As there are currently limited data available for Aboriginal Australians to be used as a reference for health-based research, a study in partnership with two Aboriginal Australian populations was carried out. The first population living at the edge of the Western Desert in Western Australia (WA) includes 72 Aboriginal Australians and has been previously described^3^. The second population includes 50 Aboriginal Australians from the Northern Territory (NT) of Australia selected as healthy individuals representative of populations studied in a recent genome-wide association study of rheumatic heart disease (RHD)^4^. WES of these individuals was carried out to provide a reference data set for known and novel variants (i.e. those exclusive to these Aboriginal Australian populations). In parallel, we updated our variant calling pipeline in line with current developments in the field^5^, and have re-called variants from the WA population to provide an improved and more concise set of variants. Together these two population data sets expand our Aboriginal Australian reference panel for use in the health sector, with particular reference to rare variants that will inform the diagnosis of rare genetic diseases in Aboriginal Australians^6^. In addition, our original dataset^3^ has been used to undertake the first systematic assessment of blood group antigen profiles in Indigenous Australian which is expected to guide transfusion practice for Aboriginal Australians^7^. For these health-based applications our data have some advantages over published^8^ WGS data for Aboriginal Australians where small numbers of DNAs (*n* of 5 to 13) from geographically and ethnically disparate groups across Australia were employed.

**Fig. 1:**
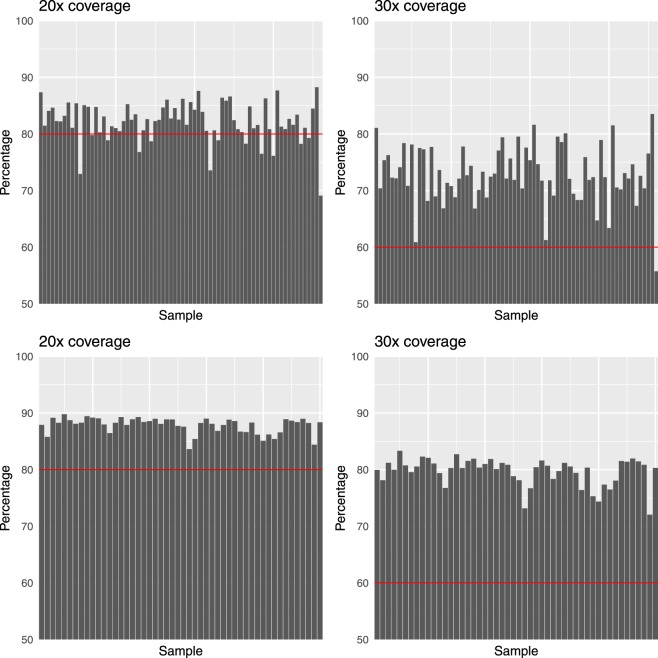
Whole exon coverage statistics. Panels show coverage at 20X and 30X for the WA population (**a,b**) and 20X and 30X coverage for the NT population (**c,d**). Each bar represents an individual sample and the percentage of bases with at least 20X or 30X coverage. Red lines mark 80% and 60% coverage at 20X and 30X depths, respectively, which all NT samples and most WA samples achieve.

The exome data were processed with GATK 4.0.2.0^9,10^ and SAMtools 1.7^11^ using an ‘intersect-then-combine’ (ITC) approach, where variant calling was performed with GATK following the best practices, and also performed with SAMtools using the mpileup function, and only variants identified by both methods were retained. The NT population had an average sequence depth of 89.8% at 20X coverage and 83.3% at 30X coverage. The WA population had an average sequence depth of 82.2% at 20X coverage and 72.9% at 30X coverage (Fig. 1).

The GATK pipeline involved several quality recalibration steps to ensure the quality of the variants called. The base quality score recalibration (BQSR) step is designed to detect systematic errors by the sequencer when it is calling base quality in the pre-processing stage. Later, the variant quality score recalibration (VQSR) calculates a new quality score named the VQSLOD (for variant quality score log-odds), which allows variant filtering with a balance of sensitivity and specificity to identify real variants whilst limiting false variants.

**Fig. 2:**
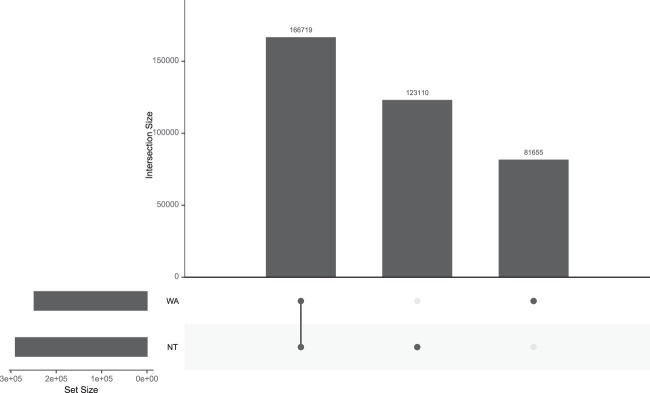
Matrix layout for the intersections of variant identified in the WA and NT populations relative to the hg19 human reference genome. Dark circles in the matrix indicate sets that are part of the intersection.

Sequences were aligned to the hg19 reference human genome and a total number of 289,829 variants were identified in at least one individual in the NT cohort and 248,374 variants in at least one individual in the WA cohort. Of these, 166,719 variants were present in both cohorts, whilst 123,110 variants were private to the NT cohort and 81,655 were private to the WA cohort (Fig. 2).

The variants identified in these cohorts were compared to single nucleotide variants (SNVs) recorded in the database for nonsynonymous SNVs’ functional predictions (dbNSFP v3.5)^2,12^. This dbNSFP is a database of human SNPs that includes 20 functional prediction scores as well as 6 conservation scores and allele frequency data from 5 datasets for the 82,832,027 nonsynonymous SNVs (nsSNVs) and splice‐site variants (ssSNVs) in the human genome. We identified 8,801 and 12,363 nsSNVs not present in dbNSFP in the WA and NT population respectively. Of these variants, 2,356 were present in both populations.

**Fig. 3:**
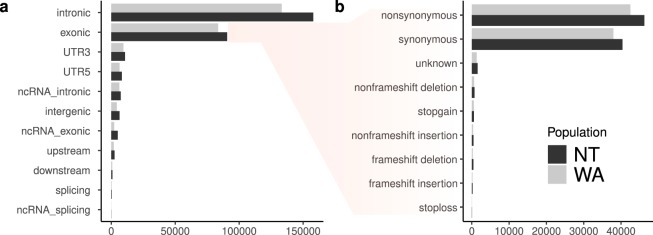
Annotation of identified variants **(a)** and consequence **(b)** for the WA and NT populations.

Variants were annotated with ANNOVAR^13^ to provide gene names and variant types and consequences (Fig. 3). The majority of variants were located in introns and exons for both populations. Totals of 83,585 and 90,642 exonic variants were present for the WA and NT populations, respectively. Whilst exons are the target regions in exome sequencing, a number of variants are also captured from outside of these target regions. A target padding of 100 bp was applied to the targets as recommended in the best practices. This target padding allows the inclusion of flanking regions so variants just outside of the target regions can be called. Exonic variants were further characterised by their functional consequence. The majority of these variants were nonsynonymous and synonymous SNVs for both populations.

## Methods

### Study populations

Subjects were recruited from Aboriginal Australian communities in the NT and in WA as described previously^3,4^. The NT individuals were selected randomly and included samples from 16 of the 19 communities originally studied^4^. All had given consent for storage and future use of deidentified DNA samples and data. The 50 individuals comprised 29 controls (11 males aged 20–58 years; 18 females aged 18–64 years) and 21 RHD cases (7 males aged 20–52 years; 14 females aged 20–60 years). These were a subset of individuals used in a genome-wide study of genetic risk factors for RHD^4^. The WA sample comprised 72 individuals from an Aboriginal Australian community of Martu ancestry for whom exome sequence data were already available as previously described^3^. This was a subset of individuals used in a genome-wide study of genetic risk factors for body mass index and type 2 diabetes^14^.

### Whole exome sequencing

The 50 NT DNA samples were prepared following the Agilent SureSelect XT Human All Exon + UTR v5 protocol and sequenced on an Illumina HiSeq. 2500 system outsourced to the Australian Genome Resource Facility (AGRF). Exome sequence data from the 72 WA samples had been similarly obtained from DNA prepared following the Illumina TruSeq protocol^3^. Sequence data for both NT and WA samples were processed using an ‘intersect-then-combine’ (ITC) approach, using GATK^9^ according to the GATK Best Practices recommendations^15,16^ and SAMtools^11^ using the mpileup function to call variants. Briefly, sequences were aligned to the hg19 reference genome with BWA-MEM^17^, followed by the removal of PCR duplicates and base quality score recalibration. Variant calling was performed using both callers and a single VCF file of the intersect was produced.

The coverage was calculated using BEDtools^18^ with the -d parameter to calculate the per ^4^base depth and then the percentage of bases with at least 20X and 30X coverage were calculated.

### Overlapping with known variants

The VCF files from both populations were intersected using BCFtools^11^; specifically using the isec command. This produced individual VCF files for variants unique to each cohort, as well as VCF files for the intersect of both cohorts.

Non-synonymous SNVs (nsSNVs) were compared to the 83,422,341 nsSNVs and splicing site SNVs (ssSNVs) present in the dbNSFP database. We excluded variants in the following publicly available databases to identify variants unique to the Aboriginal Australian populations: dbSNP 150^19^, 1000 Genomes Phase 3^20^, TWINSUK^21^, ESP6500^22^, ExAC^23^ and gnomAD^24^.

### Variant annotation

Variant annotation was performed using ANNOVAR^13^ (version 2018Apr16) to annotate variants. Specifically, the table_annovar.pl program was used to annotate variants against RefSeq sequences (version 20170601). Variants were annotated as exonic, splicing, ncRNA, UTR5, UTR3, intronic, upstream, downstream or intergenic. Exonic variants are further categorised as stoploss, stopgain, frameshift insertion, frameshift deletion, nonframeshift insertion, nonframeshift deletion, nonsynonymous SNV and synonymous SNV or unknown.

## Data Records

The full set of variants for each population has been recorded as a single multi-sample VCF file. These files have been deposited in the EGA under the accession number EGAS00001003745^25^.

## Technical Validation

The transition/transversion (Ts/Tv) ratio was calculated for each sample using BCFtools, specifically the stats function, as a quality control metric. Transitions are interchanges between purines (A, G) or pyrimidines (C, T), and transversions are changes between purines and pyrimidines. These changes occur at a ratio of 0.5 when there is no bias towards either purines or pyrimidines. In human DNA, transitions are more frequently observed due to molecular mechanisms, such as tautomeric shifts^26^, that lead to a bias in transversions of purines versus pyrimidines and as such, a ratio greater than 0.5 is expected.

**Fig. 4:**
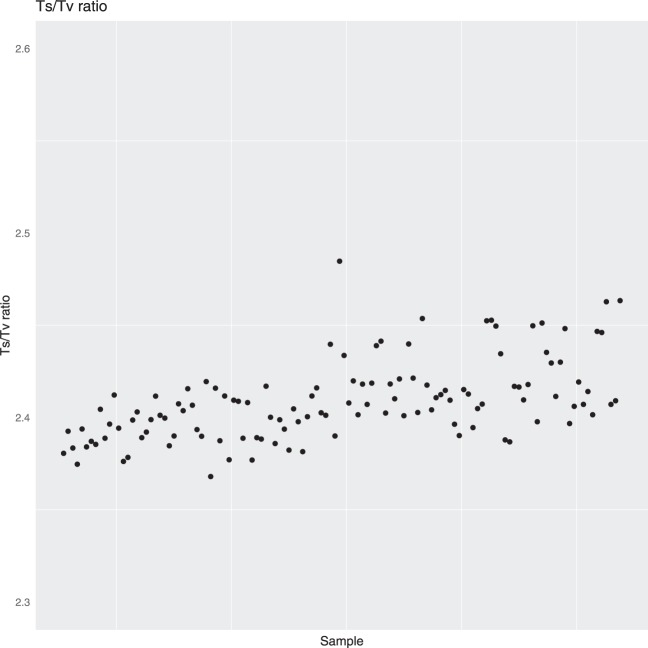
Ts/Tv ratio calculated individually for all individuals using SNVs passing the VQSR threshold. The first 50 samples on the X-axis are the NT samples, the remainder are the WA samples. The differences in average Ts/Tv ratios between NT and WA samples reflects differences in the exonic/intronic sequence ratios in the two different capture panels employed for WES.

It has been reported that a Ts/Tv ratio of 2.8 is expected for WES^27^, however this varies greatly by genome region and functionality^28^. It has been reported to be around 3.0 for exome regions, and about 2.0 outside of exome regions^29^. An average Ts/Tv ratio of 2.42 and 2.39 was observed for the WA and NT populations respectively (Fig. 4). When examining only the exonic variants, the Ts/Tv ratio was 3.01 and 3.02 for the WA and NT populations respectively, and 2.17 for non-exonic variants in both data sets, which is consistent with reported Ts/Tv ratios^29^. The Ts/Tv ratios presented are below the expected value of 2.8, however this is due to the large number of non-exonic variants captured. Whilst the target regions are exonic, the target probes have been designed to capture regions slightly outside of the exons to capture non-coding regions such as the 5′ and 3′ UTRs, which may have functional implications. Variants in these UTRs are not as constrained as those in exons, and as a result, transversions are more common and can lower the Ts/Tv ratio.

## Usage Notes

The NT study was undertaken with ethical approval from the Human Research Ethics Committee (HREC) of the Northern Territory Department of Health and Menzies School of Health Research (ID HREC-2010-1484) and the Central Australian HREC (ID HREC-2014-241). The study was overseen by a project steering committee and 3 subcommittees: Aboriginal governance, clinical, and scientific, as previously described^4^. The individual consent incorporated an “opt-in” design where participants selected which components of the study they were comfortable to participate in, and they were able to withdraw from the study at any stage. This included an option to accept or refuse continued use of their genetic or clinical data in further studies. All participants studied here accepted continued use of their genetic data. The vcf file for deidentified post-quality control data has been lodged in the European Genome-phenome Archive (accession number EGAS00001003745).

Ethical approval for the WA study was obtained from the Western Australian Aboriginal Health Ethics Committee (Reference 227 12/12). This ethics committee is responsible for reviewing health and medical research undertaken in Western Australian Aboriginal communities. It is registered with the National Health and Medical Research Council’s (NHMRC’s) Australian Health Ethics Committee (AHEC) and operates in accordance with the NHMRC National Statement on Ethical Conduct in Human Research 2007. The vcf file for de-identified data for 72 exomes re-analysed here has been lodged in the European Genome-phenome Archive (accession number EGAS00001003745).

The data for both NT and WA studies are made available through the European Genome-phenome Archive (EGA), subject to review by a study-specific Data Access Committee (DAC). This DAC is chaired by a leading Clinical Geneticist from Genetic Services of Western Australia, with membership including the Head of Aboriginal Research at the Telethon Kids Institute (TKI), the Associate Director for Aboriginal Programs at the Menzies School of Health Research, the Head of the Chronic & Severe Diseases Research Focus Area at TKI and Clinical Lead and Co-Director for Diabetes and Obesity Services at the Perth Children’s Hospital, the Director of the Office of Public Health Genomics for the WA Health Department, a leading Genetic Statistician from TKI, and a Senior Research Fellow in Aboriginal Health at TKI. The DAC is managed *ex officio* by a genetic ethicist. Access to data will be granted to qualified researchers for appropriate health related uses. A qualified researcher refers to a scientist, who is employed, or a student enrolled at, or legitimately affiliated with an academic, non-profit or government institution, or a commercial company performing Aboriginal health related diagnostic services. Applicants are asked to complete a basic application form (which includes a brief summary of the proposal, so that the DAC can determine if the planned usage falls within consents) and to agree to the terms and conditions laid out in the Data Access Agreement (DAA). The DAA must be signed by the applicant and the relevant Head of Department, Head of Institute, or equivalent. If applications include a named collaborator then the collaborator’s Institution must sign and submit a separate Data Access Agreement. Review by the DAC takes 2–3 weeks and if the application is approved, access via the EGA is then arranged for the applicant (https://www.ebi.ac.uk/ega/about/access). The application form, data access agreement, and further information are available from our website: https://www.telethonkids.org.au/aghs.

Permission to lodge de-identified genotype and basic demographic data (broad geographical location, age, sex and phenotype information) in the EGA was obtained from the Board of the local Aboriginal Health Service in WA, and from the project steering committee in NT. It should be noted that these approvals are for use of the data in health-based research, and not for use in pure population genetics research. The data are provided as reference data for health-based research and translation in Aboriginal Australian communities.
